# Associations between Body Mass Index and Probable Sarcopenia in Community-Dwelling Older Adults

**DOI:** 10.3390/nu15061505

**Published:** 2023-03-21

**Authors:** Molly Curtis, Lauren Swan, Rebecca Fox, Austin Warters, Maria O’Sullivan

**Affiliations:** 1 Department of Clinical Medicine, School of Medicine, Trinity Centre for Health Sciences, St. James’s Healthcare Campus, Trinity College Dublin, D08 W9RT Dublin, Ireland; 2 Older Person Services, Dublin North City and County Community Health Organisation, The Health Service Executive, D09 C8P5 Dublin, Ireland

**Keywords:** sarcopenia, probable sarcopenia, older adults, body mass index, malnutrition, obesity, hand grip strength

## Abstract

(1) Background/Objectives: The relationship between body mass index (BMI) and probable sarcopenia, a precursor to sarcopenia diagnosis, is unclear. While low BMI has been associated with sarcopenia risk, some evidence suggests that obesity may confer protection. We aimed to investigate the association between probable sarcopenia and BMI and, furthermore, to explore associations with waist circumference (WC). (2) Methods: This cross-sectional study included 5783 community-dwelling adults (mean age 70.4 ± 7.5 years) from Wave 6 of the English Longitudinal Study of Ageing (ELSA). Probable sarcopenia was defined using the European Working Group on Sarcopenia in Older People (EWGSOP2) criteria for low hand grip strength and/or slow chair rise. Associations between BMI and probable sarcopenia were examined using multivariable regression analysis and were similarly performed for WC. (3) Results: Our overall findings show that an underweight BMI was significantly associated with an increased likelihood of probable sarcopenia [OR (CI) 2.25 (1.17, 4.33), *p* = 0.015]. For higher BMI categories, the findings were conflicting. Overweight and obesity were associated with an increased likelihood of probable sarcopenia when defined by lower limb strength alone, [OR (CI), 2.32 (1.15, 4.70), *p* = 0.019; 1.23 (1.02, 1.49), *p* = 0.35, and 1.49 (1.21, 1.83), *p* < 0.001, respectively]. In contrast, overweight and obesity appeared protective when probable sarcopenia was assessed by low hand grip strength alone [OR (CI) 0.72 (0.60, 0.88), *p* = 0.001, and 0.64 (0.52, 0.79), *p* < 0.001, respectively]. WC was not significantly associated with probable sarcopenia on multivariable regression analysis. (4) Conclusion: This study supports the evidence that low BMI is associated with an increased likelihood of probable sarcopenia, highlighting an important at-risk group. The findings for overweight and obesity were inconsistent and may be measurement dependent. It seems prudent that all older adults at risk of probable sarcopenia, including those with overweight/obesity, are assessed to prevent underdetection of probable sarcopenia alone or with the double burden of obesity.

## 1. Introduction

By 2050, it is estimated that approximately 20% of the global population will be over the age of 60 [1]. Consequently, core elements of National and International health policies include enabling older people to remain healthy and independent for as long as possible [1]. An important aspect of enabling independence is maintaining skeletal muscle function [2]. Sarcopenia is a muscle disease characterized by an accelerated loss of muscle strength, mass, and function [2], and its incidence increases with age. The condition results in an increased risk of falls, functional decline, disability, and mortality along with a reduced quality of life [2,3,4,5,6]. The concept of ‘probable sarcopenia’, introduced by the European Working Group on Sarcopenia in Older People (EWGSOP2) in 2018, is defined by the presence of low muscle strength alone [2]. This can be assessed using simple measures of muscle strength, such as hand grip strength or chair rise tests [2], making the detection of sarcopenia in community-dwelling older populations more straightforward. Importantly, the identification of probable sarcopenia is sufficient evidence to initiate treatment, through physical activity and dietary approaches [2,3]. Thus, the concept of probable sarcopenia is pragmatic and applicable to large populations due to its ease of use as both a screening tool and a basis for intervention.

The prevalence of probable sarcopenia among community-dwelling older adults is relatively common, with estimates ranging from 19% to 34% [7,8,9,10,11]. Recognized risk factors for probable and confirmed sarcopenia include older age, physical inactivity, and comorbidity [7,8,9,12,13,14]. Older adults with malnutrition or low BMI are at increased risk of sarcopenia [15,16]. However, for probable sarcopenia the evidence is less clear. In a cross-sectional analysis of the Irish Longitudinal Study on Ageing, underweight BMI was not identified as a risk factor for probable sarcopenia, although few participants were underweight [9]. Thus, while underweight BMI is consistently identified as a determinant of confirmed or diagnosed sarcopenia [12,17,18], the relationship between BMI and probable sarcopenia has not been fully elucidated. This is particularly the case for higher BMI categories, including overweight and obesity, where the reported research into probable sarcopenia is inconsistent [7,8,10].

Previously, we reported that overweight and obesity were associated with lower odds of probable sarcopenia, which was assessed by hand grip strength only [9]. Similarly, an analysis from the Brazilian Longitudinal Study of Ageing (ELSI-Brazil) showed that BMI was inversely associated with muscle weakness measured by hand grip strength [19]. In contrast, others report an increased risk of sarcopenia/probable sarcopenia in older adults with obesity [20]. A further consideration is that the loss of muscle mass in aging is frequently offset by increases in fat mass, meaning that BMI may therefore remain unchanged [21]. Assessment of waist circumference (WC) as a clinical indicator of central obesity may provide further insight into associations between BMI and probable sarcopenia. Recent research has highlighted that an increased WC was associated with stronger hand grip strength cross-sectionally, but over an eight-year follow-up was associated with an accelerated decline in hand grip strength [22].

Both probable sarcopenia and obesity are prevalent in older populations, and each is independently associated with adverse health outcomes [23]. This issue is complex and compounded by evidence that obesity may be protective against sarcopenia and probable sarcopenia. A better understanding of these areas is important to ensure appropriate prevention, detection, and treatment for probable sarcopenia in older populations and with co-existing obesity.

In the present study, we aimed to investigate the association between probable sarcopenia and BMI in a large sample of community-dwelling older adults from the English Longitudinal Study of Ageing and to examine whether the mode of assessment of muscle strength influenced the findings. In addition, we explored if WC would provide further insight into BMI and probable sarcopenia findings. We hypothesized that probable sarcopenia would be significantly associated with BMI categories and with WC.

## 2. Materials and Methods

**Figure 1:**
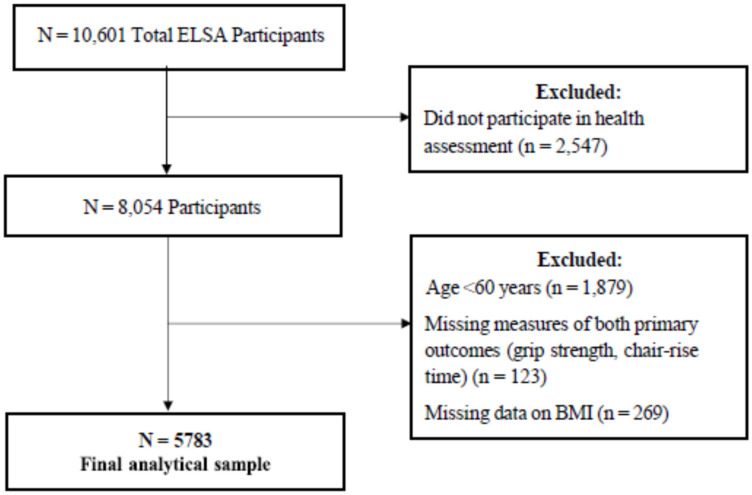
Inclusion criteria for the study group.

This study is a cross-sectional analysis of Wave 6 of the English Longitudinal Study of Ageing (ELSA), an ongoing study of community-dwelling adults aged ≥50 years in England. Full details are reported elsewhere [24]. In brief, Wave 6 took place in 2012–2013 and included 10,601 participants [24]. Data were collected through interviews and self-completion questionnaires, with 8054 completing a health assessment of physical function [25]. ELSA was conducted in line with the Declaration of Helsinki and was approved by the London Multicenter Research Ethics Committee. Written ethical consent was obtained for all waves and components of ELSA, according to the ethical approval system in operation at the time [24]. The inclusion criteria for the present study were as follows: adults > 60 years, who participated in the health assessment, with recorded data for hand grip strength, chair rise test, and BMI (Figure 1).

### 2.1. Determining Probable Sarcopenia

In accordance with the EWGSOP2 criteria, probable sarcopenia was defined as weak hand grip strength (males: <27 kg; females: <16 kg), and/or time to complete five chair rises of >15 s [2]. Hand grip strength was measured using a Smedley dynamometer [25]. Three measures were taken per hand, and the maximum score for the dominant hand was used in the analysis [7]. For the chair rise test, participants were asked to stand up and down from a firm chair, as quickly as possible, without using their arms [25]. Time taken to complete five rises was recorded. Participants deemed unable to complete the test without using their arms, or who did not attempt the test because they felt unsafe, were assumed to have probable sarcopenia [7].

### 2.2. BMI and Waist Circumference

Weight was measured without shoes and in light clothing, using Tanita™ electronic scales [24]. Height was measured using a stadiometer with the head in the Frankfurt plane [25]. BMI was categorized according to the World Health Organisation (WHO) definition [26], as underweight (<18.5 kg/m^2^), healthy (18.5–25 kg/m^2^), overweight (25–30 kg/m^2^), and obese (≥30 kg/m^2^). BMI was further analysed using a cut-off of <20 kg/m^2^ for underweight [27,28]. WC was measured using a flexible metric tape at the midpoint between the iliac crest and the last rib [24]. The mean of two valid measurements was included in the analysis. WC was grouped into metabolic risk categories as low-risk (males: <94 cm; females: <80 cm), medium-risk (males: 94–102 cm; females: 80–88 cm), and high-risk (males: ≥102 cm; females: 88 cm) [25,29,30].

### 2.3. Covariates

Demographic characteristics included sex (male; female), ethnicity (white; non-white), and age. Participants ≥ 90 years old were coded as 90 to avoid disclosure. Educational attainment was used as a marker of socioeconomic position [31]. Potential risk factors for probable sarcopenia and obesity were selected based on current evidence. In line with previous studies, comorbidities were defined using the Functional Comorbidity Index (FCI), which was modified by adding the presence of self-reported physician-diagnosed conditions to generate a score (0–8) [9]. For the purpose of the analysis, the number of conditions was then categorized as 0, 1, or 2 or more. Osteoarthritis was analysed separately due to its associations with probable sarcopenia [7]. Diabetes and cardiovascular disease (CVD) were also analysed separately, given their associations with obesity [32]. CVD was classified as any self-reported physician-diagnosed heart condition including angina, heart attack, congestive heart failure, heart murmur, abnormal heart rhythm, stroke, or other heart disease [33,34]. The number of falls in the last two years and difficulty with one or more activities of daily living (ADLs) or instrumental activities of daily living (IADLs) were self-reported [6,8]. Physical activity level was based on self-reported participation in mild, moderate, or vigorous activities at least once a week [35]. Smoking status and weekly frequency of alcohol intake were self-reported [36]. Information on alcohol history was unavailable.

## 3. Statistical Analysis

Participant characteristics were described using means and standard deviations or counts and percentages. Categorical variable characteristics of the probable sarcopenic and reference groups were compared using chi-square tests, and continuous variables were compared using independent t-tests. In multivariable models, we adjusted for covariates using backwards stepwise logistic regression. Adjusted odds ratios (OR) and 95% confidence intervals (CI) were derived. Analyses were performed using IBM SPSS Statistics, version 28.0.

## 4. Results

### 4.1. Characteristics of the Study Population Overall and by Probable Sarcopenia

The characteristics of the study population overall and according to probable sarcopenia are outlined in Table 1. Participants (*n* = 5783) were a mean age of 70.4 ± 7.5 years and 54.6% were female. Overweight and obesity (73.4%) along with increased WC (79.5%) were prevalent. Overall, 31.8% of the study population met the criteria for probable sarcopenia.

Participants with probable sarcopenia had a significantly higher frequency of underweight BMI based on both the <18.5 and <20 kg/m^2^ cut-off criteria at 1.6% and 3.9%, respectively, compared with the reference group. However, it is important to note that <4% of participants were underweight in this population. In the probable sarcopenia group, there was a significantly higher proportion of obesity (34.1% vs. 29.2%) but fewer overweight (40.0% vs. 43.8%). In addition, a greater proportion met the criteria for high-risk WC in the probable sarcopenia group compared with the reference group. With respect to other health and lifestyle characteristics, participants with probable sarcopenia were significantly older, more physically inactive, and experienced more chronic conditions, previous falls, and difficulty with ADLs/IADLs than the reference group (Table 1).

### 4.2. Associations between Probable Sarcopenia and BMI Based on Regression Analysis

Probable sarcopenia was defined by the EWGSOP2 criteria in all regression models. In Model 1, muscle strength was assessed by hand grip strength and/or chair rise test, in Model 2 by hand grip strength alone, and in Model 3 by the chair rise test alone (Table 2).

In a multivariable analysis, older adults with underweight BMI had a 2.25-fold increased likelihood of probable sarcopenia [OR 2.25, CI 1.17, 4.33, *p* = 0.015] compared with those within a healthy BMI range (model 1), with similar findings reported for Model 3. When captured by hand grip strength alone (model 2), associations between probable sarcopenia and underweight BMI were not noted.

Both overweight and obese BMI was associated with significantly greater odds of probable sarcopenia detected by slow chair rise (model 3) [OR, CI, 1.23 (1.02, 1.49), *p* = 0.35, and 1.49 (1.21–1.83), *p* < 0.001, respectively]. Conversely, overweight and obesity were associated with reduced odds of probable sarcopenia (Model 2), as detected by hand grip strength alone [OR, CI 0.72 (0.60, 0.88), *p* = 0.001, and 0.64 (0.52, 0.79), *p* < 0.001, respectively]. Elevated BMI was not significantly associated with probable sarcopenia in Model 1.

Underweight, overweight, and obese BMI were consistently associated with an increased likelihood of probable sarcopenia in Model 3 (chair rise test), with divergent findings for Model 2 (hand grip strength). In addition, all three regression models provided further evidence of a higher likelihood of probable sarcopenia associated with older age, low physical activity, lower educational attainment, chronic conditions, osteoarthritis, recurrent falls, and difficulty with ADLs or IADLs.

Finally, regression analysis for WC (Table 3) was not statistically significant overall in a model controlled for other covariates (model 1). Increased odds of probable sarcopenia was suggested for high-risk WC measurements in the lower limb assessment model, in contrast to reduced odds in the hand grip strength sarcopenia model and, importantly, the latter was not statistically significant.

## 5. Discussion

Probable sarcopenia, defined by low muscle strength [2], is a practical measure to apply in population settings. Determinants of probable sarcopenia include older age, physical inactivity, and co-morbidity [7,8,9], but findings for BMI remain inconsistent particularly for overweight and obesity [9]. In the present study, we investigated associations between BMI and probable sarcopenia in a large population of community-dwelling older adults (*n* = 5783) with a mean age of 70.4 ± 7.5 years. An underweight BMI was significantly associated with increased odds of probable sarcopenia. BMI in the overweight or obese category was not significantly associated with probable sarcopenia in the overall model, however, the results differed according to the mode of assessment employed for low muscle strength in further regression models.

The association between low BMI and increased likelihood of probable sarcopenia is consistent with evidence that an underweight BMI may be a marker of malnutrition in older adults [27,28,37]. The observations for underweight BMI were noted in the bivariate analysis, in the overall and lower limb strength regression models, but not in the hand grip strength model. Collectively, the finding suggests that older adults with underweight BMI would benefit from screening for probable sarcopenia in addition to malnutrition screening, with appropriate interventions if indicated. Indeed, low BMI, low skeletal muscle mass, or muscle strength (when mass cannot be readily assessed) are among the recommended phenotypic criteria for malnutrition by the Global Leadership Initiative on the Malnutrition (GLIM) working group [37].

Overweight and obese BMI was not significantly associated with probable sarcopenia when controlled for known risk factors in the overall model, with divergent findings based on the mode of sarcopenia assessment employed (Models 2 and 3). In this regard, overweight and obesity were significantly associated with an increased likelihood of probable sarcopenia as defined by lower limb strength alone. Conversely, higher BMI suggested protective effects when probable sarcopenia was defined by low hand grip strength alone. Previous studies have reported obesity as a risk factor for sarcopenia; recently, Crovetto Mattassi et al. [22] highlighted that participants with obesity had a 3.2 times greater risk of presenting with sarcopenia (probable and severe sarcopenia combined) compared with healthy nutritional status in a relatively small study sample. Much of the published evidence appears to favour inverse associations between probable sarcopenia risk and overweight/obesity, which is in agreement with the finding from our hand grip strength model.

An analysis of the Irish Longitudinal Study on Ageing, which employed hand grip strength only, found that overweight and obesity were associated with lower odds of probable sarcopenia [9]. Consistent with this, in the Brazilian Longitudinal Study of Ageing, obesity was inversely associated with hand grip strength [19]. Others similarly observed that a larger overall body mass, indicated by higher BMI, was associated with stronger hand grip strength [38]. Recently, obesity accompanying probable sarcopenia defined by hand grip strength showed favourable trends for frailty, compared with probable sarcopenia alone [39]. Though other findings are more complex, for example, in the Korean Frailty and Ageing Cohort Study, high BMI was not associated with muscle strength (using hand grip strength cut-offs equating to probable sarcopenia) but appeared protective against low muscle mass [40]. Of note, the latter study had a modest sample size and included community and residential care participants. The trajectory of probable sarcopenia with obesity and their combined impact on health outcomes remains to be clarified.

Indeed, the proposition that overweight or obesity might confer health benefits in older populations fits with the obesity paradox [41]; for example, a higher BMI may mitigate against unintentional weight loss or reflect fewer chronic conditions. The association between higher hand grip strength values and obesity may be explained by a greater muscle mass [40], which was not assessed in the present study. It is likely that overweight and obesity may not necessarily protect against probable sarcopenia, but rather, the finding is in part related to the mode of detection when applying hand grip strength only in assessment. The identification of probable sarcopenia by hand grip strength alone may underdetect probable sarcopenia in older adults with overweight and obesity [42]. Positive cross-sectional associations between greater muscle strength and obesity may reverse over periods of time, as noted for longitudinal WC findings [22]. BMI may also remain stable on follow-up, while muscle-related parameters decline [43].

To expand the findings beyond BMI, we included waist circumference as a measure of central obesity. On analysis, this parameter was not significantly associated with probable sarcopenia in the overall model and did not substantially add to the BMI results. Keevil et al. reported that a high WC was associated with lower grip strength [38]. Other authors observed that abdominal obesity, defined by higher WC, was associated with stronger hand grip strength at baseline in older adults, but this effect was not maintained over time and was associated with accelerated muscle strength decline in men over 8 years of follow-up [22]. Further research into probable sarcopenia and WC is warranted.

The present study supports previously identified risk factors for probable sarcopenia, namely older age, low physical activity, socioeconomic disadvantage, chronic conditions, recurrent falls, and difficulty with ADLs or IADLs [6,7,8,9,11]. Although there is a lack of consistency around BMI as a determinant of probable sarcopenia, there is a growing body of evidence around established risk factors for probable sarcopenia.

Both overweight and probable sarcopenia are public health issues prevalent across older populations. Based on the current evidence, it cannot necessarily be assumed that older people with overweight or obesity are protected from sarcopenia. Probable sarcopenia may coexist with low BMI and increase the risk of poor outcomes. Equally, probable sarcopenia and obesity may coexist, and each increases the risk of poor health outcomes [44]. Obesity is characterized by low-grade inflammation and insulin resistance which negatively impact skeletal muscle mass. A systematic review and meta-analysis [45] demonstrated that older adults with sarcopenic obesity were at increased risk of adverse musculoskeletal outcomes compared with individuals with obesity, sarcopenia, or neither condition. Treating diagnosed sarcopenia with obesity is more complex and may be associated with poorer outcomes [45], which needs further exploration in probable sarcopenia. There is ongoing work to examine the complex condition of sarcopenic obesity, its definitions, clinical relevance, and the most effective prevention and treatment strategies [23].

The present study has a number of strengths. It is based on an analysis of a large robust nationally representative sample of community-dwelling older adults in England [24], with objective health measures, including markers of obesity and muscle strength. Limitations are acknowledged, such as the cross-sectional nature of the study meaning that we cannot infer causality nor predict the impact of BMI on probable or diagnosed sarcopenia trajectories over time. BMI as a measure has inherent limitations and does not reflect weight history, unintentional weight loss, body composition, or muscle parameters. Moreover, relatively few participants were classified with underweight BMI in this ELSA population. Non-participation in health assessments, such as BMI and muscle strength measures, may under-represent those with poorer health or reduced mobility and impact the generalisability Sof the findings. Notably, the population was not ethnically diverse as previously reported [11]. Further research is needed into the detection of probable sarcopenia, its longer-term trajectory, and optimal management in community-dwelling older adults with overweight and obesity.

## 6. Conclusions

In conclusion, underweight BMI was significantly associated with an increased likelihood of probable sarcopenia in a large cohort of community-dwelling older adults, representing an important at-risk group for screening and intervention. The findings for overweight and obese BMI were conflicting and appeared, at least in part, to be measurement dependent. Overweight and obese BMI was consistently associated with an increased likelihood of probable sarcopenia, determined by lower limb strength, but protective when determined by hand grip strength. The lack of consistency suggests that high BMI values alone may be a poor determinant of probable sarcopenia. It seems sensible that all older adults at risk of probable sarcopenia should be assessed, regardless of BMI, including those with overweight or obesity, to enable early detection and treatment. Moreover, there is a need to prevent the under-detection of probable sarcopenia alone or with the double burden of obesity. Further longitudinal research is needed to understand probable sarcopenic obesity, its trajectory, and impact on health outcomes [23].

## Figures and Tables

**Table 1:** Characteristics of the Study Population Overall and Based on the Presence of Probable Sarcopenia ^a^.

	Overall Study Population (*n* = 5783)	Reference ^b^ No Probable Sarcopenia (*n* = 3945)	Probable Sarcopenia (*n* = 1838)	*p*-Value ^c^
Age, years, mean ± SD	70.4 ± 7.5	68.4 ± 6.4	74.6 ± 8.1	<0.001 *
Age categories, *n* (%)				
60–64	1539 (26.6%)	1294 (32.8%)	245 (13.3%)	<0.001 *
65–69	1470 (25.4%)	1169 (29.6%)	301 (16.4%)	<0.001 *
70–74	1073 (18.6%)	727 (18.4%)	346 (18.8%)	0.745
75–79	941 (16.3%)	512 (13.0%)	429 (23.3%)	<0.001 *
>80	760 (13.1%)	243 (6.2%)	517 (28.1%)	<0.001 *
Sex, *n* (%)				
Male	2628 (45.4%)	1884 (47.8%)	744 (40.5%)	<0.001 *
Female	3155 (54.6%)	2061 (52.2%)	1094 (59.5%)	<0.001 *
Ethnicity, *n* (%)				
White	5649 (97.7%)	3862 (97.9%)	1787 (97.2%)	0.138
Non-White	134 (2.3%)	83 (2.1%)	51 (2.8%)	0.138
Educational attainment, *n* (%)				
No formal qualification	1556 (26.9%)	861 (21.9%)	695 (37.8%)	<0.001 *
Lower sec/international qual.	1678 (29.1%)	1141 (29.0%)	537 (29.2%)	0.865
Upper secondary	1495 (25.9%)	1089 (27.7%)	406 (22.1%)	<0.001 *
Degree	1046 (18.1%)	847 (21.5%)	199 (10.8%)	<0.001 *
BMI (kg/m^2^), mean ± SD	28.2 ± 5.1	28.1 ± 4.8	28.6 ± 5.7	<0.001 *
BMI (WHO criteria), *n* (%)				
Underweight (<18.5 kg/m^2^)	53 (0.9%)	24 (0.6%)	29 (1.6%)	<0.001 *
Healthy (18.5–25 kg/m^2^)	1487 (25.7%)	1041 (26.4%)	446 (24.3%)	0.092
Overweight (25–30 kg/m^2^)	2464 (42.6%)	1728 (43.8%)	736 (40.0%)	0.008 *
Obese (≥30 kg/m^2^)	1779 (30.8%)	1152 (29.2%)	627 (34.1%)	<0.001 *
BMI (alternative criteria), *n* (%)				
Underweight (<20 kg/m^2^)	169 (2.9%)	97 (2.5%)	72 (3.9%)	0.003 *
Healthy (20–25 kg/m^2^)	1371 (23.7%)	968 (24.5%)	403 (21.9%)	0.032 *
Overweight (25–30 kg/m^2^)	2464 (42.6%)	1728 (43.8%)	736 (40.0%)	0.008 *
Obese (≥30 kg/m^2^)	1779 (30.8%)	1152 (29.2%)	627 (34.1%)	<0.001 *
Waist circumference (cm), mean ± SD	96.61 ± 13.51	96.04 ± 13.16	97.87 ± 14.17	<0.001 *
Waist circumference categories, *n* (%)				
Low-risk	1178 (20.6%)	853 (21.8%)	325 (17.9%)	<0.001 *
Medium-risk	1431 (25.0%)	1028 (26.2%)	403 (22.2%)	0.001 *
High-risk	3121 (54.5%)	2037 (52.0%)	1084 (59.8%)	<0.001 *
Waist-to-height ratio, mean ± SD	0.59 ± 0.08	0.58 ± 0.08	0.60 ± 0.08	<0.001 *
Waist-to-height ratio categories, *n* (%)				
No increased-risk	767 (13.4%)	572 (14.6%)	195 (10.8%)	<0.001 *
Increased-risk	2658 (46.4%)	1938 (49.5%)	720 (39.7%)	<0.001 *
Very high-risk	2305 (40.2%)	1408 (35.9%)	897 (49.5%)	<0.001 *
Smoking status, *n* (%)				
Never smoked	2072 (35.8%)	1464 (37.1%)	608 (33.1%)	0.003 *
Past smoker	3147 (54.4%)	2117 (53.7%)	1030 (56.0%)	0.097
Current smoker	564 (9.8%)	364 (9.2%)	200 (10.9%)	0.054
Physical activity level, *n* (%)				
Low	1341 (23.2%)	538 (13.6%)	803 (43.7%)	<0.001 *
Intermediate	2781 (48.1%)	2023 (51.3%)	758 (41.2%)	<0.001 *
High	1661 (28.7%)	1384 (35.1%)	277 (15.1%)	<0.001 *
Chronic conditions, *n* (%)				
0	1567 (27.1%)	1307 (33.1%)	260 (14.1%)	<0.001 *
1	1962 (33.9%)	1437 (36.4%)	525 (28.6%)	<0.001 *
≥2	2254 (39.0%)	1201 (30.4%)	1053 (57.3%)	<0.001 *
Cardiovascular disease, *n* (%)	1348 (23.3%)	710 (18.0%)	638 (34.7%)	<0.001 *
Diabetes, *n* (%)	677 (11.7%)	336 (9.3%)	311 (16.9%)	<0.001 *
Osteoarthritis, *n* (%)	1723 (29.8%)	991 (25.1%)	732 (39.8%)	<0.001 *
Number of falls in last 2 years, *n* (%)				
0	4199 (72.7%)	3029 (76.8%)	1170 (63.8%)	<0.001 *
1	921 (15.9%)	603 (15.3%)	318 (17.3%)	0.051
≥2	657 (11.4%)	312 (7.9%)	345 (18.8%)	<0.001 *
Difficulty with ADLs or IADLs, *n* (%)	1496 (25.9%)	602 (15.3%)	894 (48.6%)	<0.001 *

Notes: ^a^ Data are presented as frequency (%) or mean ± standard deviation. ^b^ Did not meet the criteria for probable sarcopenia based on the EWGSOP2 cut-offs for low grip strength and/or slow chair rise. ^c^ Chi-squared X^2^ and independent t-test used for comparison between probable sarcopenic group and reference group (* *p* < 0.05). Abbreviations: ADLs, activities of daily living; BMI, body mass index; IADLs, instrumental activities of daily living; International qual., international qualification; Lower sec., lower secondary; *n*, frequency; SD, standard deviation; WHO, World Health Organisation.

**Table 2:** Multivariable regression analysis for BMI and other health factors associated with probable sarcopenia in community-dwelling older adults.

Probable Sarcopenia									
		HGS and/or CRT Model 1			HGS Only Model 2			CRT Only Model 3	
Variable	OR	95% CI for OR	* *p*-value	OR	95% CI for OR	* *p*-value	OR	95% CI for OR	* *p*-value
Age, years	1.09	1.08–1.10	<0.001	1.10	1.08–1.11	<0.001	1.09	1.07–1.10	<0.001
Ethnicity									
White	Ref								
Non-white	1.55	1.03–2.34	0.036	-	-	-	-	-	-
Educational attainment			<0.001			0.002			0.005
No formal	1.68	1.36–2.09	<0.001	1.55	1.18–2.04	0.001	1.52	1.19–1.94	<0.001
Low secondary	1.35	1.09–1.66	0.006	1.51	1.15–1.97	0.003	1.21	0.95–1.54	0.125
Upper secondary	1.26	1.01–1.57	0.037	1.18	0.89–1.56	0.259	1.22	0.95–1.56	0.125
Degree	Ref			Ref			Ref		
BMI (WHO criteria)			0.046			<0.001			<0.001
Underweight	2.25	1.17–4.33	**0.015**	1.30	0.65–2.62	0.458	2.32	1.15–4.70	**0.019**
Healthy	Ref			Ref			Ref		
Overweight	0.92	0.78–1.09	0.322	0.72	0.60–0.88	**0.001**	1.23	1.02–1.49	**0.035**
Obese	0.94	0.78–1.12	0.475	0.64	0.52–0.79	**<0.001**	1.49	1.21–1.83	**<0.001**
Physical activity level			<0.001			<0.001			<0.001
Low	2.92	2.40–3.55	<0.001	2.57	2.02–3.29	<0.001	3.27	2.62–4.08	<0.001
Intermediate	1.32	1.12–1.56	0.001	1.36	1.09–1.71	0.007	1.38	1.13–1.69	0.002
High	Ref			Ref			Ref		
Smoking status	-	-	-	-	-	-			<0.001
Never smoker							Ref		
Past smoker							1.01	0.86–1.19	0.890
Current smoker							1.72	1.33–2.24	<0.001
Chronic conditions			<0.001			<0.001			0.010
0	Ref			Ref			Ref		
1	1.12	0.92–1.35	0.252	1.16	0.90–1.49	0.254	1.08	0.87–1.35	0.496
≥2	1.44	1.18–1.76	<0.001	1.67	1.30–2.15	<0.001	1.35	1.08–1.69	0.010
CVD	1.32	1.13–1.53	<0.001		-	-	1.38	1.17–1.62	<0.001
Diabetes	1.27	1.05–1.55	0.017	1.35	1.08–1.68	0.008		-	-
Osteoarthritis	1.36	1.17–1.58	<0.001	1.21	1.02–1.45	0.034	1.29	1.09–1.52	0.003
Falls in past 2			<0.001			<0.001			<0.001
years									
0	Ref			Ref			Ref		
1	1.13	0.94–1.35	0.190	1.09	0.88–1.34	0.452	1.18	0.97–1.44	0.091
≥2	1.74	1.42–2.13	<0.001	1.87	1.51–2.33	<0.001	2.29	1.85–2.84	<0.001
Difficulty with ADLs and IADLs	2.55	2.19–2.97	<0.001	1.67	1.40–1.99	<0.001	2.62	2.23–3.09	<0.001

Table 2 notes: Probable sarcopenia was defined by low hand grip strength (HGS) and/or slow chair rise time (CRT) (Model 1), by low HGS alone (Model 2), or slow CRT alone (Model 3). Shading denotes variables removed from the model on backward stepwise regression, not significantly associated with probable sarcopenia when controlled for covariates. Multivariable logistic regression models were restricted to participants with complete co-variable information: Model 1 [*n* = 5769; 0.2% excluded due to missing data]. Model 2: [*n* = 5693; 1.6% excluded due to missing data]. Model 3: [*n* = 5494; 5% excluded due to missing data]. * *p* < 0.05 is considered to be statistically significant. CI, confidence interval; OR, odds ratio; Ref, reference category.

**Table 3:** Multivariable logistic regression models for WC and other risk factors for probable sarcopenia in community-dwelling older adults.

Probable Sarcopenia									
		HGS and/or CRT Model 1			HGS Only Model 2			CRT Only Model 3	
Variable	OR	95% CI for OR	* *p*-value	OR	95% CI for OR	* *p*-value	OR	95% CI for OR	* *p*-value
Age, years	1.09	1.08–1.10	<0.001	1.1	1.09–1.11	<0.001	1.08	1.07–1.09	<0.001
Ethnicity	Ref 1.55	1.03–2.34	0.036						
White Non-white									
Educational attainment			<0.001			<0.001			0.005
No formal	1.67	1.34–2.06	<0.001	1.62	1.23–2.13	<0.001	1.54	1.2–1.97	<0.001
Low secondary	1.34	1.08–1.65	0.008	1.54	1.18–2.03	0.002	1.24	0.97–1.58	0.089
Upper secondary	1.25	1.01–1.56	0.043	1.18	0.87–1.57	0.259	1.24	0.96–1.59	0.096
Degree	Ref			Ref			Ref		
Waist circumference						<0.001			0.009
Low-risk				Ref			Ref		
Medium-risk				0.79	0.63–1.00	0.054	1.13	0.89–1.43	0.307
High-risk				0.61	0.50–0.76	<0.001	1.35	1.10–1.66	0.005
Physical activity level			<0.001			<0.001			<0.001
Low	2.93	2.41–3.56	<0.001	2.67	2.09–3.43	<0.001	3.24	2.59–4.06	<0.001
Intermediate	1.32	1.12–1.56	0.001	1.42	1.13–1.78	0.002	1.39	1.14–1.70	0.001
High	Ref			Ref			Ref		
Smoking status, *n* (%)									<0.001
Never smoker							Ref		
Past smoker							1.01	0.86–1.18	0.925
Current smoker							1.69	1.30–2.19	<0.001
Chronic conditions			<0.001			<0.001			0.006
0	Ref			Ref			Ref		
1	1.13	0.93–1.36	0.219	1.18	0.91–1.51	0.207	1.1	0.88–1.37	0.4
≥2	1.46	1.20–1.78	<0.001	1.68	1.31–2.17	<0.001	1.38	1.10–1.74	0.005
CVD	1.32	1.13–1.53	<0.001				1.38	1.17–1.63	<0.001
Diabetes	1.26	1.04–1.53	0.021	1.35	1.08–1.69	0.008			
Osteoarthritis	1.35	1.16–1.57	<0.001	1.23	1.03–1.47	0.024	1.27	1.07–1.50	0.005
Falls in past 2			<0.001			<0.001			<0.001
years									
0	Ref			Ref			Ref		
1	1.12	0.94–1.34	0.204	1.11	0.90–1.37	0.343	1.17	0.96–1.43	0.117
≥2	1.74	1.42–2.12	<0.001	1.91	1.54–2.38	<0.001	2.35	1.89–2.91	<0.001
Difficulty with ADLs and IADLs	2.54	2.18–2.95	<0.001	1.65	1.38–1.97	<0.001	2.64	2.25–3.11	<0.001

Table 3 notes: Probable sarcopenia was defined by low hand grip strength (HGS) and/or slow chair rise time (CRT) (Model 1), by low HGS alone (Model 2), or slow CRT alone (Model 3). Shading denotes variables removed from the model on backward stepwise regression, not significantly associated with probable sarcopenia when controlled for covariates. Multivariable logistic regression models were restricted to participants with complete co-variable information: Model 1 [*n* = 5769; 0.2% excluded due to missing data]. Model 2: [*n* = 5640. 2.5% excluded due to missing data]. Model 3: [*n* = 5447; 5.8% excluded due to missing data]. * *p* < 0.05 is considered to be statistically significant.

## Data Availability

The English Longitudinal Study of Ageing datasets are available to Researchers through the UK Data Service at https://beta.ukdataservice.ac.uk/datacatalogue/series/series?id=200011#!/access-data and can be downloaded by registering and accepting an End User License.
